# Colonoscopy screening for colorectal cancer in Egypt: a nationwide cross-sectional study

**DOI:** 10.1186/s12885-024-11828-3

**Published:** 2024-01-25

**Authors:** Abdallah R. Allam, Mostafa A. Elsayed, Ibrahim Tawfiq Daghash, Ali M. Abdelaziz, Omnia M. Mostafa, Hamdy Khaled Sabra, Ahmed Monib Eldaboush, Noor Maged Badrawy Ahmed, Rawan Tarek Elweza, Enas Sherif Adwy, Abdelrahman Elbendary Hammad, Ibrahim Ali Kabbash, Ahmed Hafez Allam, Ammar Ayman Bahbah, Marwa Ibrahim Ewis, Mohamed Mohamed Shawqi, Mostafa B. Behery, Yara Mohamed El-Said, Ahmed Eid Radwan, Mahmoud T. KhallafAllah, Omar Ali Aboshady, Mohamed A. Gouda

**Affiliations:** 1 Faculty of Medicine, Menoufia University, Menoufia, Egypt; 2 Faculty of Medicine, Alexandria University, Alexandria, Egypt; 3 Faculty of Medicine, South Valley University, Qena, Egypt; 4 Faculty of Medicine, Tanta University, Tanta, Egypt; 5 Faculty of Medicine, Al-Azhar University, Cairo, Egypt; 6 Faculty of Medicine, Ain Shams University, Cairo, Egypt; 7 Faculty of Medicine, Mansoura University, Dakahlia, Egypt; 8 Faculty of Medicine, Benha University, Cairo, Qalubia Egypt; 9 Faculty of Medicine, Cairo University, Cairo, Egypt; 10 College of Pharmacy, Purdue University, West Lafayette, IN USA

**Keywords:** Colorectal cancer, Screening, Colonoscopy, Egypt

## Abstract

**Background:**

Current guidelines advocate for colorectal cancer (CRC) screening in adults who are at risk by using direct visualization methods such as colonoscopy. However, in Egypt, there is a paucity of data regarding the current practice of colonoscopy screening. Moreover, more information is needed about the knowledge and attitudes of potential participants regarding the procedure and possible barriers that can limit their participation.

**Methods:**

We conducted a nationwide cross-sectional study using an interview-based survey of patients aged 45 years or above who presented to outpatient clinics of nine university hospitals throughout Egypt. Participants were surveyed to assess their compliance with CRC colonoscopy screening guidelines, their knowledge of and attitude towards colonoscopy screening, and their perspective on potential barriers to colonoscopy screening.

**Results:**

A total of 1,453 participants responded to our survey in the nine study centers. Only a minority of participants (2.3%) were referred for CRC screening. Referral rates were higher among those who knew someone with a history of CRC (5.3% vs 1.5%, *p* < 0.001) or had a discussion with their physician about CRC (25.8% vs 0.7%, *p* < 0.001). Few responders (3.2%) had good knowledge regarding CRC screening. After introducing the concept of CRC screening to all participants, most patients (66.7%) showed a positive attitude towards having the procedure. Financial burden and fear of results were the two most frequently cited barriers to undergoing CRC screening (81.1%; and 60.1%, respecteively).

**Conclusions:**

Despite the positive attitude, there is insufficient knowledge about CRC screening among eligible participants in Egypt. This has probably contributed to low compliance with current CRC screening guidelines and needs to be addressed at the national level.

**Supplementary Information:**

The online version contains supplementary material available at 10.1186/s12885-024-11828-3.

## Introduction

Colorectal cancer (CRC) is the third most common cancer worldwide and the second most common cause of cancer-related mortality [1]. Survival rates vary significantly according to the disease stage at which cancer was diagnosed. For example, the 5-year survival rate dramatically increases to 90% when patients are diagnosed at an early stage compared to 14% in patients with metastatic stage at presentation [2, 3]. Efforts have, therefore, been made to empower CRC early detection through the implementation of screening programs in different countries. In the United States, such efforts led to a significant reduction in CRC-related mortality [4–6].

Current guidelines advocate for CRC screening in adults aged 45–75 years using direct visualization methods such as colonoscopy or stool-based tests such as fecal occult blood test (FOBT) [7, 8]. However, participation rates and physicians’ referrals remain a significant challenge despite the enormous efforts put in that regard [9]. For example, nearly 30% of eligible adults in the United States are not compliant with the current recommendations for CRC screening [10]. A study in Hungary reported that a similar percentage of participants did not even hear about CRC screening methods [11]. A third study in the West Pacific Region reported a low level of patient awareness of the disease burden and poor adherence to screening [12]. The frequency of non-compliance is reported to be even higher in developing countries either due to lack of awareness [13], patients’ refusal due to cultural barriers, financial burden [14], or non-referral by healthcare providers [15].

In Egypt, data about the prevalence of CRC are not consistent but it is estimated to rank as the eighth most common cancer diagnosis in some reports [1, 16]. The incidence rate is estimated to be 9.8 per 100,000 cases [17]. However, there is a paucity of relevant studies assessing the current attitudes towards CRC screening and its actual practice. The Egyptian insurance system provides coverage for CRC screening in eligible adults [18] but compliance with standard guidelines remains unknown. It is noteworthy that compliance with other screening programs including breast cancer remains relatively poor [19]. Moreover, we have at least some evidence suggesting that most CRC cases in Egypt are diagnosed in late stages [20] which casts doubts on the current practice of CRC screening without the possibility of reaching definitive conclusions.

In this study, we aimed to evaluate the current practice of CRC screening in Egypt and compliance with the most recent guidelines. Moreover, we aimed to assess the knowledge about CRC screening, explore patients’ attitudes, and investigate potential barriers.

## Methods

### Study setting

**Fig. 1:**
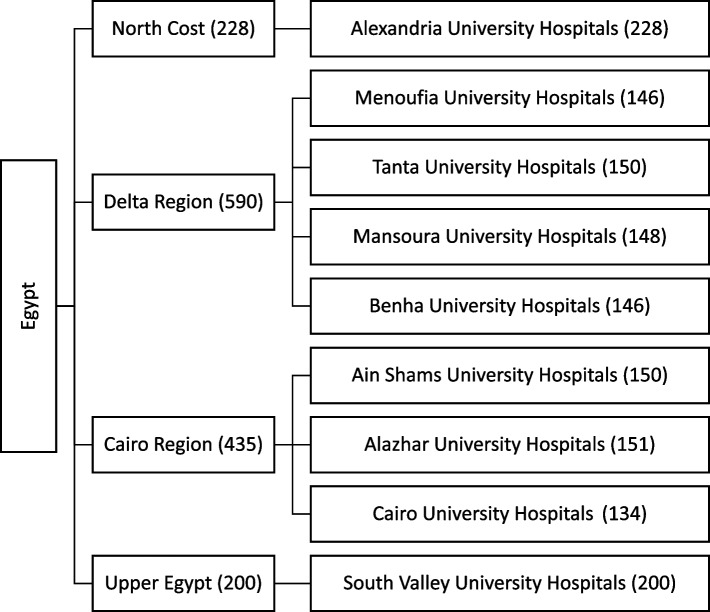
Study allocation strategy

We conducted a nationwide cross-sectional study in Egypt to assess the practice of CRC screening from patients’ perspective. The study was done in nine centers geographically distributed all over Egypt (North cost – Delta region – Cairo region—Upper Egypt) using proportionate allocation that was based on the Egyptian population census (Fig. 1) [21]. Further details on the included study centers are provided in the supplement (Supplementary Table 1). Ethical approval was obtained from the Tanta University Faculty of Medicine prior to starting data collection. Participation was voluntary and informed consent was obtained prior to starting data collection.

### Study population and sample

In this study, we included patients aged 45 and above who attended general medicine outpatient clinics in the included study centers. Patients who have been diagnosed with any type of cancer including CRC and patients presenting with gastrointestinal-related symptoms (e.g. constipation) were excluded.

### Questionnaire development

A questionnaire was specifically developed for the purpose of this study and used during the interview for data collection. Our primary outcome was to evaluate the current practice of CRC screening in Egypt from patients’ perspective. After a thorough literature review, the study team developed an initial draft; which was discussed, and reviewed by three experts. The draft was then pilot-studied on 20 patients to assess its clarity, accuracy, and duration of completion. The final version of the questionnaire (Additional file 2) was developed based on the pilot study results. Patients included in the pilot study were excluded from the final analysis.

The questionnaire included five sections (sociodemographic data, knowledge, attitude, barriers, and practice of CRC screening). The knowledge section included closed-ended questions and self-reported scales to assess participants’ self-perceived and actual knowledge. Four knowledge questions were used to characterize good and bad knowledge including the need for screening in asymptomatic individuals, age at CRC screening initiation, frequency of CRC screening, and tools used in CRC screening. Participants with correct knowledge in three or more components were considered to have good knowledge. Similarly, the attitudes section included closed-ended and scale questions for defining participants’ attitudes toward CRC screening, where scores of six or more (on a scale of 10) were considered positive. Prior to the assessment of attitudes, which was only done after the assessment of knowledge, an introduction was given about CRC screening to reduce the effect of knowledge deficiency on attitudes. Additionally, participants answered close-ended questions to assess whether they had been referred for screening by their doctors and whether they had undergone colonoscopy for CRC screening. The questionnaire also comprehensively assessed the barriers to CRC screening including lack of awareness, personal fears, or healthcare-related barriers from the patients’ perspectives.

### Data collection

The questionnaire was administered to the target population in their own language during medical visits to hospitals’ clinics. Data were collected in a structured interview format in a private area within the waiting areas of outpatient clinics.

### Statistical analysis

Data were analyzed using IBM SPSS Statistics for Windows version 28 (IBM Corp., Armonk, N.Y., USA). Categorical variables were presented as frequencies and percentages and continuous variables were presented as medians and interquartile ranges (IQR). Pearson’s Chi-square test and Fisher exact test were used to explore the associations between the categorical variables. A *p*-value of < 0.05 was considered statistically significant. To determine the appropriate sample size, Raosoft.com was utilized, aiming to achieve a 95% confidence level with a margin of error of 2.5%. The anticipated sample size was initially calculated to be 1428 participants.

## Results

### Characteristics of participants

We included 1,453 participants from nine Egyptian centers (54.1% males and 45.9% females). The median age of responders was 55 years (IQR; 49, 61). Nearly half of our sample had secondary education or higher (49.7%); and the majority were from rural areas (60.7%). Almost one-fifth of the included participants (19.6%) knew someone who was previously diagnosed with CRC and 15.8% knew a patient with CRC who had a disease-related death (Table 1).

**Table 1:** Characteristics of participants

Variables	Number (%)
Age	55 (12)
Sex	
Males	786 (54.1%)
Females	667 (45.9%)
Residency	
Urban	571 (39.3%)
Rural	882 (60.7%)
Education	
Post-graduate	48 (3.3%)
College	292 (20.1%)
Secondary school	382 (26.3%)
Primary school	388 (26.7%)
Uneducated	343 (23.6%)
Currently employed	
Yes	755 (52%)
No (including retired)	694 (47.8%)
Income level (self-reported)	
Low income	871 (59.9%)
Middle income	564 (38.8%)
High income	18 (1.2%)
Method of Payment of Medical Bills	
Health insurance (covered by my employer)	72 (5%)
Government Support	595 (40.9%)
Out of Pocket	786 (54.1%)
Participants with relative who had pre-cancerous polyp	37 (2.5%)
Participants with relative who had CRC	96 (6.6%)
Participants who knew others diagnosed with CRC	239 (16.4%)
Participants who knew anyone who died of CRC	230 (15.8%)
Participants diagnosed with colorectal disease	292 (20.1%)
Participants who had discussions with their physician about cancer in general	287 (19.8%)
Participants who had discussions with their physician about cancer screening	258 (17.8%)
Participants who had discussions with their physician about CRC	89 (6.1%)
Participants who had discussions with their physician about CRC screening	62 (4.3%)

*CRC* Colorectal cancer

Data presented as median (IQR) or number (percentage)

### Knowledge

A minority of the participants (4.3%) had a previous discussion with their physicians about CRC screening. On a scale of ten, the median participants’ self-perceived knowledge level about CRC screening was 1 (IQR; 1, 2). Few responders (4.7%) had a self-perceived knowledge score of 6 or more. Using knowledge status dichotomization by points of interest, only 46 participants (3.2%) had actual good knowledge regarding CRC screening. However, most participants (80.7%; n = 1173) wanted to obtain more information about CRC screening (Table 2).

**Table 2:** Participants’ knowledge and attitude towards CRC screening

Variables		Number (%)
Knowledge		
Self-perceived knowledge about CRC screening (Scale of 10)		1 (1, 2)
Actual knowledge about CRC screening (Two groups)		
	Good knowledge	46 (3.2%)
	Poor knowledge	1407 (96.8%)
Participants who knew the start age for CRC screening		181 (12.5%)
Participants who knew the frequency for CRC screening		97 (6.7%)
Participants who knew the tool for CRC screening		93 (6.4%)
Participants who agreed that people should be screened for CRC even without experiencing symptoms		684 (47.1%)
Participants who agreed that early-stage CRC can be asymptomatic		618 (42.5%)
Participants who wanted to obtain more information about CRC screening		1173 (80.7%)
Attitude		
Attitude towards CRC screening (Scale of 10)		8 (5, 10)
Attitude towards CRC screening (Two groups)		
	Positive attitude	967 (66.6%)
	Negative attitude	486 (33.4%)
Participants who were willing to undergo screening if recommended by their doctor		909 (62.6%)
Participants who mentioned that they will only have CRC if it becomes mandatory		465 (32%)
Participants who doubted the effect of CRC screening on early detection of CRC		216 (14.9)

*CRC* Colorectal cancer

Data presented as median (IQR) or number (percentage)

Self-reported good knowledge (as demonstrated by a score of 6 or more on a 10-point scale) was higher among participants with secondary school education or higher compared to those with lower levels of education (12.6% vs. 2.2%, *p* < 0.001). Additionally, self-perceived good knowledge was more likely in people who knew someone diagnosed by CRC than those who did not (11.6% vs. 3%, *p* < ,0.001).

Using the four knowledge components described in the methods, good actual knowledge of CRC screening as assessed by selected parameters was more likely in patients with secondary education or higher compared to patients with a lower level of education (6% vs. 0.4%, *p* < 0.001). A higher proportion of participants with good knowledge was also observed in responders with high or middle income compared to those with low income (6.9% vs. 0.7%, *p* < 0.001). Moreover, good knowledge was more likely in participants who knew someone diagnosed with CRC (8.1% vs. 2%, *p* < 0. 001), or died of CRC (7% vs. 2.5%, *p* < 0.001), or had a prior diagnosis of colorectal disease (7.9% vs 2%, *p* < 0.001). Participants with good knowledge were also more likely to have had a discussion with their physician about CRC (32.6% vs. 1.2%, *p* < 0.001), cancer screening in general (15.1% vs. 0.6%, *p* < 0.001), or CRC screening (38.7% vs. 1.6%, *p* < 0. 001) (Table 3).

**Table 3:** Associations between different studied variables and participants knowledge and attitude towards CRC screening

	Knowledge			Attitude		
	Good	Poor	*P* value	Positive	Negative	*P* value
Sex						
Males	28 (3.6%)	758 (96.4%)	0.349	574 (73%)	212 (27%)	** < 0.001**
Females	18 (2.7%)	649 (97.3%)		393 (58.9%)	274 (41.1%)	
Residency						
Urban	22 (2.5%)	860 (97.5%)	0.069	466 (81.6%)	105 (18.4%)	** < 0.001**
Rural	24 (4.2%)	547 (95.8%)		501 (56.8%)	381 (43.2%)	
Education						
High level	43 (6%)	679 (94%)	** < 0.001**	542 (75.1%)	180 (24.9%)	** < 0.001**
Low level	3 (0.4%)	728 (99.6%)		425 (58.1%)	306 (41.9%)	
Currently employed						
Yes	34 (4.5%)	721 (95.5%)	**0.003**	563 (74.6%)	192 (25.4%)	** < 0.001**
No (including retired)	12 (1.7%)	682 (98.3%)		400 (57.6%)	294 (42.4%)	
Income level						
Middle or high	40 (6.9%)	542 (93.1%)	** < 0.001**	468 (80.4%)	114 (19.6%)	** < 0.001**
Low	6 (0.7%)	865 (99.3%)		499 (57.3%)	372 (42.7%)	
Do you know any relative with pre-cancerous polyp?						
Yes	6 (16.2%)	31 (83.8%)	0.001*	33 (89.2%)	4 (10.8%)	**0.003**
No	40 (2.8%)	1376 (97.2%)		934 (66%)	482 (34%)	
Do you know any relative with CRC?						
Yes	8 (8.3%)	88 (91.7%)	0.009*	88 (91.7%)	8 (8.3%)	** < 0.001**
No	38 (2.8%)	1319 (97.2%)		879 (64.8%)	478 (35.2%)	
Do you know others diagnosed with CRC?						
Yes	21 (8.8%)	218 (91.2%)	** < 0.001**	198 (82.8%	41 (17.2%)	** < 0.001**
No	25 (2.1%)	1189 (97.9%)		769 (63.3%	445 (36.7%)	
Do you know anyone died of CRC?						
Yes	16 (7%)	214 (93%)	** < 0.001**	189 (82.2%)	41 (17.8%)	** < 0.001**
No	30 (2.5%)	1193 (97.5%)		778 (63.6%)	445 (36.4%)	
Have you been diagnosed with colorectal disease?						
Yes	23 (7.9%)	269 (92.1%)	** < 0.001**	232 (79.5%)	60 (20.5%)	** < 0.001**
No	23 (2%)	1138 (98%)		735 (63.3%)	426 (36.7%)	
Have you had discussions with your physician about cancer in general?						
Yes	40 (13.9%)	247 (86.1%)	** < 0.001**	233 (81.2%)	54 (18.8%)	** < 0.001**
No	6 (0.5%)	1160 (99.5%)		734 (63%)	432 (37%)	
Have you had discussions with your physician about cancer screening?						
Yes	39 (15.1%)	219 (84.9%)	** < 0.001**	210 (81.4%)	48 (18.6%)	** < 0.001**
No	7 (0.6%)	1188 (99.4%)		757 (63.3%)	438 (36.7%)	
Have you had discussions with your physician about CRC?						
Yes	29 (32.6%)	60 (67.4%)	< 0.001*	78 (87.6%)	11 (12.4%)	** < 0.001**
No	17 (1.2%)	1347 (98.8%)		889 (65.2%)	475 (34.8%)	
Have you had discussions with your physician about CRC screening?						
Yes	24 (38.7%)	38 (61.3%)	< 0.001*	56 (90.3%)	6 (9.7%)	** < 0.001**
No	22 (1.6%)	1369 (98.4%)		911 (65.5%)	480 (34.5%)	

*CRC* Colorectal cancer, bold indicates significant associations

Data presented as number (percentage)

^*^Indicates the use of Fisher Exact test

### Attitude

On a scale of ten, participants rated their agreement with the need to perform CRC screening in patients aged 45 years and above. The median score for attitude of all participants was 8 (IQR; 5, 10). Most participants (66.7%; n=967) had positive attitude towards CRC screening using status dichotomization (score ≥ 6). 62.6% of responders (n=909) demonstrated an interest in undergoing CRC screening if recommended by their doctor (Table 2).

Participants with self-reported good knowledge or actual good knowledge were more likely to have positive attitude towards CRC screening (91.2% vs. 65.3%; and 89.1% vs. 65.8%; *p* < 0.001 and *p* = 0.001; for self-perceived and actual knowledge respectively). Positive attitudes were more likely to be observed in males compared to females (73% vs. 58.9%, *p* < 0.001) and in participants who had secondary education or higher compared to those with lower levels of education (75.1% vs. 58.1%, *p* < 0.001). Additionally, positive attitudes were significantly higher in participants with high or middle income than in participants with low income (80.4% and 57.3%, *p* < 0.001), and in residents of urban areas compared to residents of rural areas (81.6% vs. 56.8%, *p* < 0.001). Moreover, positive attitudes were higher in participants who knew someone diagnosed with CRC (91.7% vs. 64.8%, *p* < 0.001) or died of CRC (82.2% vs. 63.6%, *p* < 0.001) compared to participants who did not. Responders with a history of colorectal disease or a prior discussion with their physician about CRC, cancer screening, or CRC screening also had higher rates of positive attitudes towards CRC screening compared to participants who did not (79.5% vs. 63.3%, *p* < 0.001; and 81.2% vs. 63%, *p* < 0. 001; respectively) (Table 3).

### Practice

The majority of participants in our sample were never referred for CRC screening (97.7%, n = 1419). Among 32 who were referred, only one completed the colonoscopy procedure for the purpose of CRC screening.

The referral rates for CRC screening were higher in males compared to females (3.7% vs. 0.4%, *p* < 0.001), in participants who had secondary education or higher compared to participants who had not (3.6% vs. 0.8%, *p* < 0.001), and in participants with high or middle income compared to participants with low income (3.8 vs. 1.1%, *p* = 0.001). Referral rates were higher in participants who had good knowledge and good attitude towards CRC screening (37% vs. 1.1%, *p* < 0.001; 3% vs. 0.6%, *p* = 0.003 for knowledge and attitude; respectively) (Table 4).

**Table 4:** Factors associated with participants referral rates

	Referral rates		
	Referred	Not referred	*P* value
Sex			
Males	29 (3.7%)	755 (96.3%)	** < 0.001**
Females	3 (0.4%)	664 (99.6%)	
Residency			
Urban	17 (3%)	554 (97%)	0.107
Rural	15 (1.7%)	865 (98.3%)	
Education			
High level	26 (3.6%)	695 (96.4%)	** < 0.001**
Low level	6 (0.8%)	724 (99.2%)	
Currently employed			
Yes	26 (3.5%)	727 (96.5%)	**0.001**
No (including retired)	6 (0.9%)	688 (99.1%)	
Income level			
Middle or high	22 (3.8%)	559 (96.2%)	**0.001**
Low	10 (1.1%)	860 (98.9%)	
Do you know any relative with pre-cancerous polyp?			
Yes	3 (8.1%)	34 (91.1%)	0.046*
No	29 (2.1%)	1385 (97.9%)	
Do you know any relative with CRC?			
Yes	6 (6.3%)	90 (93.8%)	**0.005**
No	26 (1.9%)	1329 (98.1%)	
Do you know others diagnosed with CRC?			
Yes	14 (5.9%)	225 (94.1%)	** < 0.001**
No	18 (1.5%)	1194 (98.5%)	
Do you know anyone died of CRC?			
Yes	14 (6.1%)	216 (93.9%)	** < 0.001**
No	18 (1.5%)	1203 (98.5%)	
Have you been diagnosed with colorectal disease?			
Yes	17 (5.8%)	275 (94.2%)	** < 0.001**
No	15 (1.3%)	1144 (98.7%)	
Have you had discussions with your physician about cancer in general?			
Yes	26 (9.1%)	261 (90.9%)	** < 0.001**
No	6 (0.5%)	1158 (99.5%)	
Have you had discussions with your physician about cancer screening?			
Yes	26 (10.1%)	232 (89.9%)	** < 0.001**
No	6 (0.5%)	1187 (99.5%)	
Have you had discussions with your physician about CRC?			
Yes	23 (25.8%)	66 (74.2%)	** < 0.001**
No	9 (0.7%)	1353 (99.3%)	
Have you had discussions with your physician about CRC screening?			
Yes	22 (35.5%)	40 (64.5%)	< 0.001*
No	10 (0.7%)	1379 (99.3%)	
Knowledge			
Actual knowledge about CRC screening			
Good knowledge	17 (37%)	29 (63%)	** < 0.001**
Poor knowledge	15 (1.1%)	1390 (98.9%)	
Participants who knew the start age for CRC screening			
Correct	16 (8.8%)	165 (91.2%)	< 0.001*
Incorrect	16 (1.3%)	1254 (98.7%)	
Participants who knew the frequency for CRC screening			
Correct	13 (13.4%)	84 (86.6%)	< 0.001*
Incorrect	19 (1.4%)	1335 (98.6%)	
Participants who knew the tool for CRC screening			
Correct	19 (20.4%)	74 (79.6%)	< 0.001*
Incorrect	13 (1%)	1345 (99%)	
Participants who approved people should be screened for CRC even without experiencing symptoms			
Yes	25 (3.7%)	659 (96.3%)	** < 0.001**
No	7 (0.9%)	760 (99.1%)	
Participants who approved that early-stage CRC can be asymptomatic			
Yes	26 (4.2%)	590 (95.8%)	** < 0.001**
No	6 (0.7%)	829 (99.3%)	
Participants who wanted to obtain more information about CRC screening			
Yes	31 (2.6%)	1140 (97.4%)	**0.019**
No	1 (0.4%)	279 (99.6%)	
Attitude			
Attitude towards CRC screening			
Positive attitude	29 (3%)	936 (97%)	**0.003**
Negative attitude	3 (0.6%)	483 (99.4%)	
Are you willing to undergo screening if recommended by your doctor			
Yes	26 (2.9%)	881 (97.1%)	**0.027**
No	6 (1.1%)	538 (98.9%)	
I will only have CRC if it becomes mandatory			
Yes	6 (1.3%)	459 (98.7%)	0.103
No	26 (2.6%)	960 (97.4%)	
I doubt the effect of CRC screening on early detection of CRC			
Yes	5 (2.3%)	211 (97.7%)	0.805*
No	27 (2.2%)	1208 (97.8%)	

*CRC* Colorectal cancer, bold indicates significant associations

Data presented as number (percentage)

^*^Indicates the use of Fisher Exact test

### Barriers

**Fig. 2:**
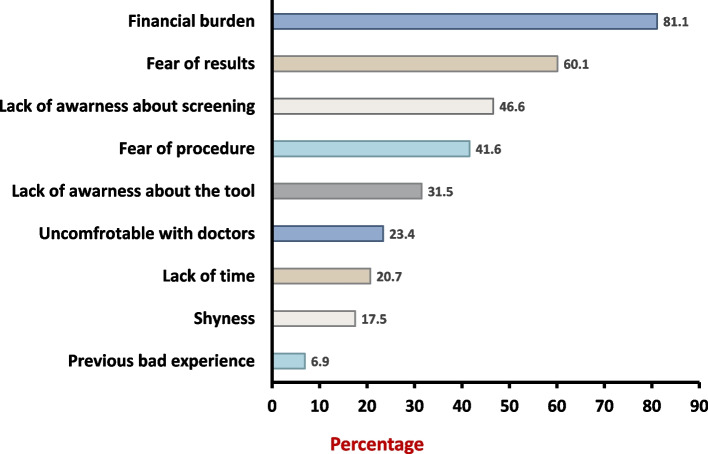
Barriers towards colorectal cancer screening

Financial burden and fear of results were the most frequently reported barriers to undergoing CRC screening (reported in 81.1% and 60.1% of participants, respectively). Other barriers included lack of awareness about CRC screening (46.6%), fear of the procedure (41.6%), discomfort associated with seeking elective health care services (23.4%), lack of time (20.7%), and shyness (17.5%). When asked to specify only one barrier, almost half of our sample (47.9%) chose financial burden as the most crucial barrier (Fig. 2).

Participants who reported financial burden as a barrier were more likely to be males (85% vs. 76.5%, *p* < 0.001), employed (83.3% vs. 78.8%, *p* < 0.001), residents of urban areas (86.5% vs. 77.6%, *p* < 0.001), and had known someone diagnosed with CRC (87.4% vs. 79.5%, *p* < 0.001), or died of CRC (87.8% vs. 79.8%, *p* < 0.001). Fear of results was significantly more frequent in participants with secondary school education or higher (63% vs. 57.2%, *p* = 0.023), residents of rural areas (63.4% vs. 55%, *p* = 0.001), participants who had previous discussions with their physicians about cancer, CRC, or CRC screening (71.1% 57.4%, *p* < 0.001), and participants with negative attitudes (74.9% vs. 52.6%, *p* < 0.001).

### Questionnaire impact

To assess whether the questionnaire delivery could have influenced participants’ attitudes towards undergoing CRC screening, we asked participants about their willingness to undergo CRC screening before and after completing the questionnaire. Initially, only 2.4% (35 participants) considered undergoing CRC screening. After the interview, the proportion of participants willing to undergo CRC screening increased to 64.3% (*n* = 935).

## Discussion

The magnitude of CRC in Egypt is exacerbated by the late diagnosis of cases which is in turn associated with poor prognosis [20]. Our study highlights the low rates of referrals to CRC screening in Egypt, which might be a contributing factor. Moreover, we demonstrate that despite positive attitudes towards CRC screening, low levels of awareness may be the key challenge that needs to be addressed by policymakers and healthcare providers.

Using a nationwide survey, we found that only 2.3% of eligible participants were referred for CRC screening. This rate is much lower than that reported in other parts of the world. For example, in the United States, more than two-thirds of the eligible adults are currently undergoing screening. In Canada and France, 37% and 59% of eligible adults had undergone screening, respectively [10, 22, 23]. However, our results are comparable to those of other studies from the Middle East, including Lebanon (15%) and Saudi Arabia (8.6%) [13, 14]. Similarly, low rates of participation have been reported in other developing countries including India (1.5%) and Indonesia (3%) [12]. The low participation rates in our study could be attributed to a lack of knowledge regarding CRC risk factors and screening guidelines. Our findings also revealed that referral rates were higher in participants who had discussions with their physicians about cancer screening in general or CRC screening in particular. Personal experience with someone diagnosed with or deceased from CRC was also associated with higher referrals for screening.

Among the surveyed participants, we found that only 3.2% of participants had good knowledge of CRC screening guidelines, indicating notably low awareness among the Egyptian population. In comparison, other countries in the Middle East and Europe had higher levels of awareness. For example, 38.3% and 45% of participants in Lebanon and Saudi Arabia, respectively, had good knowledge about CRC screening [24, 25]. Studies in other parts of the world have also reported similarly fair levels of knowledge about CRC screening [11, 12]. This disparity might be attributed to the lack of accessible knowledge resources and awareness campaigns.

In our study, many factors have been associated with participants’ level of knowledge, including educational level, income, residency, and personal experience with CRC. These findings are consistent with prior research as individuals with higher levels of education may have a better understanding of medical concepts, while those with personal experience may have a greater appreciation for the importance of screening [26, 27]. Previous discussions with physicians about CRC or CRC screening have been shown to significantly improve the level of awareness. This aligns with previous research as physicians can provide individuals with personalized information about screening that is more likely to be trusted and acted upon [28]. Despite the low level of knowledge observed in our study, over 80% of the participants wanted to obtain more information about CRC screening showing the value of addressing existing knowledge gaps and enhancing awareness among the Egyptian population.

Most participants exhibited a positive attitude towards CRC screening (66.7%). Almost two-thirds of our participants (62.6%) were willing to undergo CRC screening if recommended by their doctors, which is similar to data from Saudi Arabia and Lebanon [14, 24]. A high educational level or knowledge of someone diagnosed with CRC had a favorable effect on our participants’ attitudes. This association has been previously reported in another study [29]; and can be linked to realizing the hazards, comorbidities, and crucial role of early detection in CRC prognosis. Interestingly, our 10-min questionnaire increased willingness to undergo screening by more than 60%. This would highlight the link between awareness and attitudes; and support calls for national awareness campaigns that could probably improve attitudes and compliance. However, this does not necessarily imply a direct causal impact of the questionnaire but rather suggests that the information contained in the questionnaire could have contributed to changing participants' perceptions about the importance of CRC screening.

Financial burden was by far the most reported barrier for not undergoing CRC screening followed by fear of results and low knowledge about CRC screening. This could be related to the overall economic state in Egypt. In comparison, fear of results and lacking of knowledge about CRC screening were the most important barriers in Saudi Arabia [13]. In our study, fear of results was higher among participants with higher levels of education and those with prior disease background either through discussions with their physicians or knowing someone diagnosed with the disease. This finding may be attributed to a greater understanding and knowledge of the disease and its potential consequences.

Our study had several limitations. First, we used a convenience sampling method to recruit participants from outpatient clinics at university hospitals, which might not be optimum. However, the large number of responders should improve the generalizability of results. Second, the analysis was constrained by the small number of participants who were referred for CRC screening. Therefore, associations with other parameters that were explored as secondary endpoints should be interpreted with caution. Moreover, surveying attitudes of participants who often visit healthcare facilities may be influenced by their health-seeking behavior. Additionally, explaining CRC screening just before asking participants about their attitudes may have not fully eliminated the impact of knowledge deficiency. Furthermore, we didn’t assess the awareness of CRC symptoms and its potential associations with our study outcomes, which could be further explored in future studies. Lastly, physicians’ perspectives on referral rates and potential barriers to CRC screening were not explored. This would be an area of interest for future studies and would complement data that we reported from patients’ perspectives. Despite these limitations, our study provides the largest to date assessment of colonoscopy screening practice in Egypt. This should help inform decision makers about possible challenges that may need to be addressed.

## Conclusion

Despite the positive attitude, there is deficient knowledge about CRC screening among eligible participants in Egypt. This has probably resulted in low compliance with current CRC screening guidelines. Implementing an organized national screening program may help to increase public awareness and promote CRC screening practice in the community.

### Supplementary Information

**Additional file 1. **Study centers overview.

**Additional file 2.** Attitude and practice towards colorectal cancer screening in Egypt: a nationwide survey.

## Data Availability

The datasets analyzed during the current study are available from the corresponding author on reasonable request.

## Abbreviation

CRC: Colorectal cancer
