# Supplementary material for: Colonoscopy screening for colorectal cancer in Egypt: a nationwide cross-sectional study
Source: BMC Cancer. 2024 Jan 25;24:131. doi: 10.1186/s12885-024-11828-3 (PMC10809530; doi:10.1186/s12885-024-11828-3)
Supplement: Supplementary file 1 — Additional file 1. Study centers overview. [file 12885_2024_11828_MOESM1_ESM.pdf]

Additional file 1: Study Centers Overview

Region	Center(s)	Number of included participants	Brief overview
**North Coast**	Alexandria University Hospital	228	Alexandria University Hospital is a medical institution located in Alexandria, Egypt. It is one of the oldest and largest hospitals in the country, providing healthcare services to the local community and international patients. The hospital contains more than 200 beds, multi-operating rooms, intensive care units and outpatient units.
**Delta Region**	Menoufia University Hospital	146	Menoufia University Hospital is a medical facility located in Egypt's Menoufia governorate. It is one of the largest medical institutions in Egypt and offers a range of medical facilities and services providing quality healthcare to patients of all ages.
	Tanta University Hospital	150	Tanta University Hospital is one of the largest teaching hospitals associated with Tanta University in Egypt, serving as a major healthcare provider for the Gharbia governorate region. It offers a wide range of medical and surgical services.
	Mansoura University Hospital	148	Mansoura University Hospital is a medical facility located in Dakahial governorate, Egypt. It is affiliated with Mansoura University and offers a wide range of medical and surgical services to the local community.
	Benha University hospital	146	Benha University Hospital is a public hospital located in Qalyuba governorate, Egypt. It is affiliated with Benha University and provides medical care to the local community.
**Cairo Region**	Ain Shams University Hospital	150	Ain Shams University Hospital is a major teaching hospital and research center Located in Cairo governorate, Egypt. It offers a wide range of medical and surgical services.

	Alazhar University Hospital	151	Alazahr University Hospital is a major teaching hospital Located in Cairo governorate, Egypt. It offers a wide range of medical and surgical services.
	Cairo University Hospital	134	Cairo University Hospital is a medical facility located in Cairo, Egypt. It is one of the largest and most prestigious hospitals in the country, providing medical services to both residents and visitors. The hospital is affiliated with Cairo University and offers a wide range of medical specialties.
**Upper Egypt**	South Valley University Hospital	200	South Valley University Hospital is a major teaching hospital and research center Located in Qena governorate, Egypt. It offers a wide range of medical and surgical services.
