# Supplementary material for: Colonoscopy screening for colorectal cancer in Egypt: a nationwide cross-sectional study
Source: BMC Cancer. 2024 Jan 25;24:131. doi: 10.1186/s12885-024-11828-3 (PMC10809530; doi:10.1186/s12885-024-11828-3)
Supplement: Supplementary file 2 — Additional file 2. Attitude and practice towards colorectal cancer screening in Egypt: a nationwide survey. [file 12885_2024_11828_MOESM2_ESM.pdf]

ATTITUDE AND PRACTICE TOWARDS COLORECTAL CANCER SCREENING IN EGYPT: A NATIONWIDE SURVEY

This is a 10-minutes survey consisting of 5 main sections. Data are collected as part of a nationwide study to assess the situation of colorectal cancer screening in Egypt.

PERSONAL DETAILS
1. **Patient Name:**
2. **Age:**
3. **Gender:**<br>O Male O Female
4. **Occupation:**
5. **Highest Level of Education:**<br>O Primary School<br>O Secondary School<br>O College<br>O Postgraduate Studies
6. **Income:**<br>O High-income<br>O Middle-income<br>O Low-income
7. **Residence:**<br>O Urban O Rural
8. **How do you pay for your medical service?**<br>O I have health insurance (covered by my employer)<br>O My medical service is covered by the government<br>O I pay for my medical service from my own money

9. **Have you ever considered undergoing screening for early detection of colorectal cancer?**<br>O Yes O No

HISTORY
1. **Do you have a parent, brother, sister, or a child of yours who has ever been diagnosed with colorectal cancer?**<br>O Yes O No
2. **Do you have a parent, brother, sister, or a child of yours who has ever been diagnosed with pre-cancerous polyps?**<br>O Yes O No
3. **Apart from immediate relatives mentioned above, do you know anyone who was diagnosed with colorectal cancer?**<br>O Yes O No
4. **Do you know anyone who died of colorectal cancer?**<br>O Yes O No
5. **Have you ever been diagnosed with colorectal cancer?**<br>O Yes O No
6. **Have you ever been diagnosed with cancers other than colorectal cancer?**<br>O Yes O No
7. **Have you ever been diagnosed with any colorectal disease (other than cancer)?**<br>O Yes O No
8. **If you answered the above question by yes, what was the disease?***
9. **Have you ever had a discussion with your physician about colorectal cancer?**<br>O Yes O No
10. **Have you ever had a discussion with your physician about cancer screening?**<br>O Yes O No
11. **Have you ever had a discussion with your physician about CRC screening?**<br>O Yes O No

KNOWLEDGE ABOUT COLORECTAL CANCER SCREENING

1. **On a scale of ten, how do you rate your current knowledge of CRC screening?**
2. **In your own word, what would be a benefit from CRC screening?**
3. **At what age are you supposed to undergo colorectal cancer screening?**
4. **How frequent are you supposed to do screening after reaching the recommended age?**
5. **Do you know what are the tools that can be used for colorectal cancer screening?**
   O Yes O No
6. **If you answered the above question by yes, what are the tools you have heard about? ***
7. **Should people be screened for CRC even without experiencing any symptoms?**
   O Yes O No
8. **Can early-stage colorectal cancer be asymptomatic?**
   O Yes O No
9. **Would you like to obtain more information about CRC Screening?**
   O Yes O No

ATTITUDE TOWARDS COLORECTAL CANCER SCREENING

Screening means looking for a disease when you have no symptoms suggestive of it. It can detect precancerous conditions that require removal and follow up or can detect cancer at early stages that can have better recovery rates

1. **On a scale of ten, how much do you agree with the need for performing CRC screening in patients 45 years old and above?**
2. **Are you willing to undergo CRC screening yourself if recommended by your doctor?**
   O Yes O No
3. **I will only have CRC Screening if it becomes mandatory**
   O Yes O No
4. **I doubt the effect of CRC screening on early detection of CRC**
   O Yes O No

BARRIERS OF COLORECTAL CANCER SCREENING

In this section, we are trying to explore potential barriers that prevent people from undergoing CRC screening

1. **In your own words, what would be the most important factor preventing you from undergoing colorectal cancer screening?**
2. **Each of the following may be a factor preventing you or other people from undergoing screening. For each of them, please indicate if it is a factor for you (Y/N)?**
   O Knowledge about screening
   O Knowledge about the tool used in screening
   O Fear of the procedure used for screening
   O Financial burden
   O Fear of results
   O Previous bad experience
   O Feeling uncomfortable about dealing with doctors
   O Lack of time
   O Shyness
3. **Among the previously mentioned factors (listed below), what is the most important factor that would prevent you or other people from undergoing CRC screening?**
   O Knowledge about screening
   O Knowledge about the tool used in screening
   O Fear of the procedure used for screening
   O Financial burden

O Fear of results
O Previous bad experience
O Feeling uncomfortable about dealing with doctors
O Lack of time
O Shyness

PRACTICE OF COLORECTAL CANCER SCREENING
**1. Have you ever been recommended CRC screening?**<br>O Yes O No
**2. Have you ever had a colonoscopy (A doctor looked at your entire colon and rectum with a special instrument while you were asleep (with anesthesia). You had a liquid diet and took medication to clean out your bowels before the test)?**<br>O Yes O No
**3. If you answered the above question by yes, then for what purpose? ***
**4. If you answered the above question by yes, then when was it / the last time?***
**5. Have you ever had a fecal occult blood test (You used a flat stick to put samples of a bowel movement on a special card and returned it to your doctor or a laboratory for testing)?**<br>O Yes O No
6. **If you answered the above question by yes, then for what purpose? ***
**7. If you answered the above question by yes, then when was it / the last time? ***

A. After completing this session, are you interested in undergoing screening for early detection of colorectal cancer?
O Yes O No
